# Phylogenetic relationships among Capuchin (Cebidae, Platyrrhini) lineages: An old event of sympatry explains the current distribution of *Cebus* and *Sapajus*

**DOI:** 10.1590/1678-4685-GMB-2017-0012

**Published:** 2018

**Authors:** Antonio Marcio Gomes Martins-Junior, Jeferson Carneiro, Iracilda Sampaio, Stephen F. Ferrari, Horacio Schneider

**Affiliations:** ^1^ Instituto de Estudos Costeiros, Universidade Federal do Pará, Bragança, PA, Brazil; ^2^ Laboratório de Genética, Evolução e Bioinformática, Instituto Federal do Pará, Tucurui, PA, Brazil; ^3^ Departamento de Ecologia, Universidade Federal de Sergipe, São Cristovão, SE, Brazil; ^4^ Department of Life Sciences, Roehampton University, London, UK.

**Keywords:** Capuchins, phylogeography, phylogeny, taxonomy, biogeography

## Abstract

Capuchin monkeys are currently represented by four species of *Cebus* and eight of *Sapajus*. This group is taxonomically complex and several questions still need to be clarified. In the current study, using mtDNA markers and a larger sample representation than in previous studies, we seek to understand the phylogenetic relationships among the capuchin lineages and their historical biogeography. All 12 species of capuchins were analyzed for the mitochondrial Control Region and Cytochrome *b* to test two biogeographical hypotheses: “Reinvasion of the Amazon (ROA)” and “Sympatric Evolution (SEV)”*.* The phylogenetic relationships among distinct lineages within genera is consistent with an evolutionary diversification pattern probably resulting from an explosive process of diversification and dispersal between 2.0 Ma and 3.0 Ma. Also, the analyses show that the ancestral capuchins were distributed in a wide area encompassing the Amazon and Atlantic Forest. Our results support the SEV hypothesis, showing that the current syntopic distribution of *Cebus* and *Sapajus* can be explained by a sympatric speciation event in the Amazon. We also indicate that the recently proposed species taxonomy of *Cebus* is not supported, and that *S. cay* and *S. macrocephalus* are a junior synonym of *S. apella*.

## Introduction

The taxonomy of capuchin monkeys (*Cebus* and *Sapajus*, Cebidae) is among the most controversial of Neotropical primates (Platyrrhini). Hershkovitz (1949) proposed four species, the gracile *Cebus albifrons*, *Cebus nigrivittatus* (= *olivaceus*) and *Cebus capucinus*, and the robust *Cebus apella*, with several subspecies. Groves (2001) elevated several these subspecies to valid species, i.e., *Cebus libidinosus*, *Cebus xanthosternos* and *Cebus nigritus*. Subsequent reviews (Silva Jr, 2001; Oliveira and Langguth, 2006) added five species, *Cebus kaapori*, *Cebus macrocephalus*, *Cebus cay*, *Cebus flavius*, and *Cebus robustus*. A new taxonomic proposal for the capuchins based on a genetic and morphological interpretation was recently presented by Alfaro *et al.* (2012) referring to *Cebus* for the gracile (or untufted) capuchins and *Sapajus* for the robust (or tufted) capuchins.

**Figure 1:**
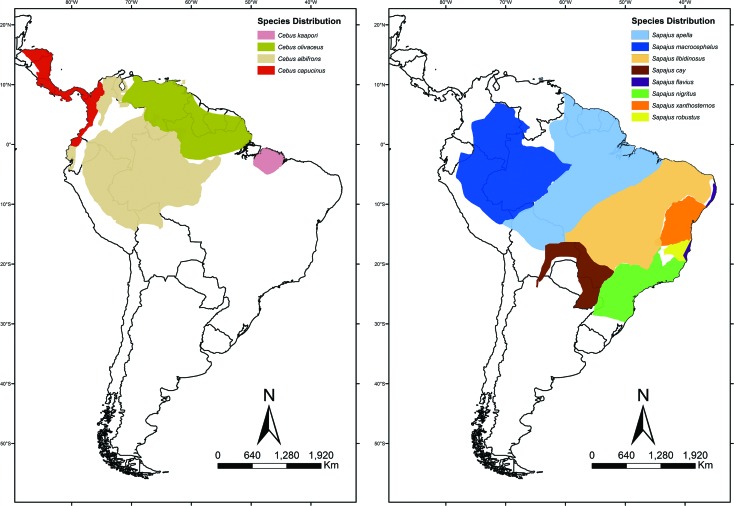
Geographical distribution of robust (*Sapajus*) and gracile (*Cebus*) capuchins species. Map constructed based on information provided by Silva Jr (2001), Lynch Alfaro *et al.* (2012), and IUCN (2016).

The gracile capuchins (*Cebus* spp.) are found from northern South America to southern Central America, whereas the robust capuchins (*Sapajus* spp.) are found throughout most of South America, as far south as northern Argentina (Figure 1). The two genera are sympatric throughout much of the Amazon basin (Silva Jr, 2001; Lynch Alfaro *et al.*, 2012; IUCN, 2016). Currently four species of *Cebus* and eight of *Sapajus* are recognized (Silva Jr, 2001; Alfaro *et al.*, 2012).

While some authors (Rosenberger, 2012; Feijó and Langguth, 2013) disagree with the *Cebus*–*Sapajus* division, Martins-Junior *et al.* (2015) supported this arrangement based on five nuclear loci, and there is a growing consensus about this taxonomic arrangement (Garbino, 2015; Schneider and Sampaio, 2015). However, the species diversity of the two genera and their origin and pattern of diversification continues to be discussed, and several species have been ressurected (Boubli *et al.*, 2012; Rylands and Mittermeier, 2013).

A number of different geological and climatic factors have been identified as drivers of the diversification of the present-day biota of South America (Haffer, 2008; Hoorn *et al.*, 2010; Ribas *et al.*, 2012), in particular the formation of forest refugia during different periods of the Pleistocene (Martins *et al.*, 2009, 2011; de Thoisy *et al.*, 2010; Ruiz-García *et al.*, 2011). Over the past 25 years, however, a growing body of evidence (e.g., Nelson *et al.*, 1990; Bush, 1994; Colinvaux *et al.*, 2000; Rull, 2008) has contradicted the Pleistocene refugia hypothesis (Ashley and Vaughn, 1995; Ribas *et al.*, 2005; Fuchs *et al.*, 2011, 2015). At the same time, an increasing number of studies have reinforced the important role of Amazonian rivers as geographic barriers in the diversification of many vertebrate groups (Hayes and Sewlal, 2004; Ribas *et al.*, 2012; Sousa-Neves *et al.*, 2013), including primates (Vallinoto *et al.*, 2006; Couette, 2007; Boubli *et al.*, 2015; Lynch Alfaro *et al.*, 2015; Mercês *et al.*, 2015).

Lynch Alfaro *et al.* (2012) concluded that the capuchins originated in the western Amazon basin approximately 6.7 million years ago (Ma). In this scenario, *Cebus* would have arisen in the northern Amazon basin 2.1 Ma, and *Sapajus* in the Brazilian Atlantic Forest or Cerrado savanna at around 2.7 Ma. These authors interpret the current sympatry of the two genera as the result of the recent reinvasion of the Amazon basin by *Sapajus* from central Brazil, explained by their “Reinvasion of the Amazon (ROA)” hypothesis. Nascimento *et al.* (2015) challenged this interpretation based on the re-analysis of the data of Lynch Alfaro *et al.* (2012), concluding that the capuchins originated in the northern Atlantic Forest. And recently, Lima *et al.* (2017) using three mitochondrial markers (Cyt *b*, Control Region and a fragment of the Cytochrome Oxidase subunit I – COI), provided support the ROA hypothesis of the origin and distribution of the capuchin monkeys in South and Central America.

Given this, the current study aimed to provide a comprehensive analysis of the geographic origins and phylogenetic relationships of the capuchins through the sequencing of two mitochondrial genes, Cytochrome *b* (Cyt *b*) and the Control Region in a broad geographic and taxonomic sample of capuchins. These two genes which have been used widely in studies of the evolution of Neotropical primates (Bonvicino *et al.*, 2001; Cortés-Ortiz *et al.*, 2003; Nascimento *et al.*, 2005; Vallinoto *et al.*, 2006; Casado *et al.*, 2010; Babb *et al.*, 2011; Matauschek *et al.*, 2011; Boubli *et al.*, 2012, 2015; Lynch Alfaro *et al.*, 2012; Mercês *et al.*, 2015).

The results obtained by Lynch Alfaro *et al.* (2012) and Lima *et al.* (2017) support vicariance, dividing ancestral capuchin populations in Amazonia versus the Atlantic Forest and a Pleistocene `Amazonian invasion’ by *Sapajus* to explain the present-day sympatry of *Cebus* and *Sapajus*. The present study intends to test this hypothesis against a new one proposed by us, which assumes that the common ancestor of all the capuchins occupied a wide distribution in different South American biomes (from the Amazon to the Atlantic Forest) and gave origin to extant *Cebus* and *Sapajus* by a sympatric speciation process.

## Materials and Methods

### Ethics statement

This research adhered to the American Society of Primatologists’ Principles for the Ethical Treatment of Primates.

### Sample collection and laboratory procedures

**Figure 2:**
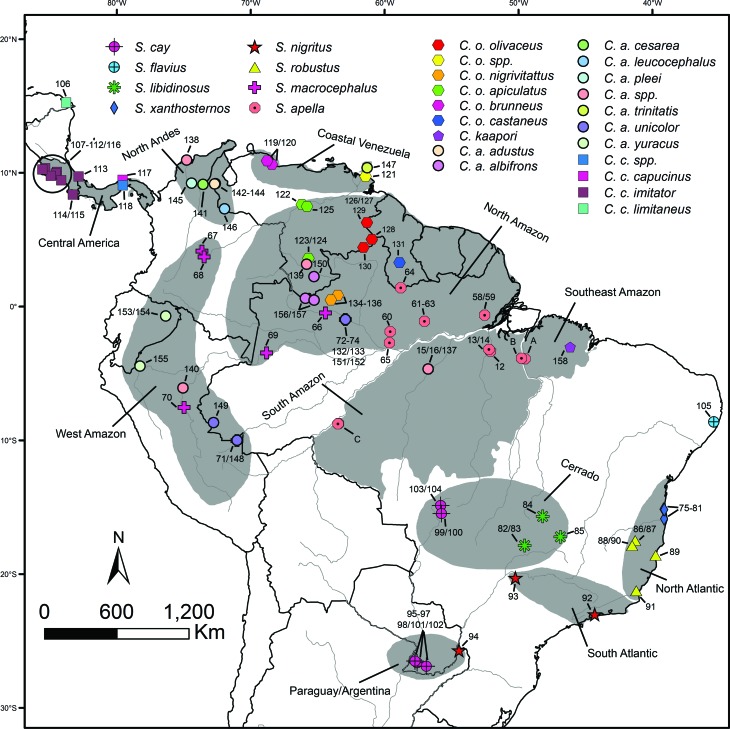
Biogeographic zones analyzed (gray spots) and the collection sites of the *Cebus* and *Sapajus* samples. A = samples 1-4; B = samples 5-11; C = 17-57.

Total DNA was extracted from blood, muscle and liver samples from 72 capuchin monkeys and purified in using the Wizard Genomic DNA Purification Kit (Promega). Most of these specimens (65) were from wild, with the remaining seven specimens provided by the captive institutions (Figure 2, Table S1). All captive animals were identified based on their morphological characteristics.

About 600 bp of the mitochondrial Control Region (HVI region and ~ 200 bp of the initial portion of the d-loop region) and the Cyt *b* gene – partial or complete – were amplified by PCR in a Verit 96 well thermocycler (Applied Biosystems). The Control Region PCR assays were carried out using primers for the *Cebus* Control Region L and *Cebus* Control Region R (Schneider *et al.*, 2012). A portion (~ 500 bp in size) and the entire of Cyt *b* gene were amplified using the primers Cytb1F and Cytb3R (Lynch Alfaro *et al.*, 2012), and MVZ05 (Irwin *et al.*, 1991) and MVZ16 (Smith and Patton, 1993), respectively. Preparation of the reactions and the PCR protocol were the same as described by Casado *et al.* (2010) for the Cyt *b* gene and Schneider *et al.* (2012) for the Control Region. Amplification products were purified and then sequenced on an Applied Biosystems 3500 XL automatic Genetic Analyzer (Life Technologies).

To check for possible amplification of *numts* rather than true mtDNA, all sequences were submitted to the BLASTn and the Cyt *b* sequences were translated. All sequences were deposited in the GenBank with codes from MF472455 to MF472591 (Table S1).

### Datasets, sequence alignment, model, and data partition selection

In addition to the sequences produced in the current study, 45 Control Region and 80 Cyt *b* gene sequences previously published by Casado *et al.* (2010), Oliveira *et al.* (2011), Boubli *et al.* (2012) and Lynch Alfaro *et al.* (2012) for capuchin monkeys were downloaded from GenBank. Sequences for different genera of Platyrrhini were also downloaded for these two mitochondrial markers.

Two datasets were used. The first dataset (DS1) was composed by 946 aligned cytochrome b mitochondrial DNA base pairs of 60 capuchin monkeys and 20 of other Neotropical primates, representing all three families (Cebidae, Atelidae and Pitheciidae), to estimate the crown age of capuchin monkeys and test monophyly. The second dataset (DS2) consisted of 1,481 base pairs of two mtDNA genes (Control Region and Cyt *b*), concatenated of 146 terminal taxa. All sequences were aligned by Clustal X (Larkin *et al.*, 2007) with default parameters and manually checked in PhyDE^®^ (Müller *et al.*, 2010).

To estimate the nucleotide substitution models and partitioning schemes that best fit each dataset, the PartitionFinder 1.1.1 (Lanfear *et al.*, 2012) software was used. Selections were made using the Bayesian Information Criteria (BIC). In the specification of subsets of alignments, the Control Region locus was defined as a single data block and the Cyt *b* gene was partitioned according with the three codon positions. All information about the datasets as length, number of samples, evolutionary models, etc. can be found in Table S2.

### Divergence time estimates among capuchin monkeys and other Platyrrhini main clades

To estimate the crown age of capuchin monkeys, we used BEAST 1.8.3 (Drummond *et al.*, 2012) software. Four calibration points based on four distinct Platyrrhini fossils were used: ^†^ *Stirtonia*, which provided a minimum age of 12.6 Ma (Hershkovitz, 1970; Kay *et al.*, 1987) for crown Atelidae; ^†^ *Proteropithecia* (Kay *et al.*, 1998), which provided a minimum age of 15.7 Ma for crown Pitheciidae; ^†^ *Neosaimiri* (Rosenberger *et al.*, 1991; Takai, 1994) provided a minimum age of 12.5 Ma for crown Cebinae; and ^†^ *Lagonimico*, which provided a minimum age of 13.4 Ma for crown Callitrichinae (Kay, 1994).

The nodes were calibrated under a non-correlated lognormal relaxed molecular clock model. The split between Catarrhini and Platyrrhini (~ 36 Ma) was used as the upper limit for the nodes calibrated under the lognormal distributions. The data was not partitioned and the HKY+I+G model was used, as selected by PartitionFinder 1.1.1 (Lanfear *et al.*, 2012).

Four independent runs of MCMC were carried out with 100,000,000 generations being sampled every 10,000 generations. The convergence of the chains and the ESS values for the different parameters were analyzed in the Tracer 1.6 software. The LogCombiner 1.8.3 software was used to combine the .log and .trees files of the four independent runs. A burn-in of 10% was used and TreeAnnotatoor 1.8.3 software, was used to summarize all nodes and the *a posteriori* distributions of each parameter in a Maximum Clade Credibility (MCC) tree. The trees were visualized in FigTree 1.4.2.

### Phylogeographic analysis in capuchin monkeys

**Figure 3:**
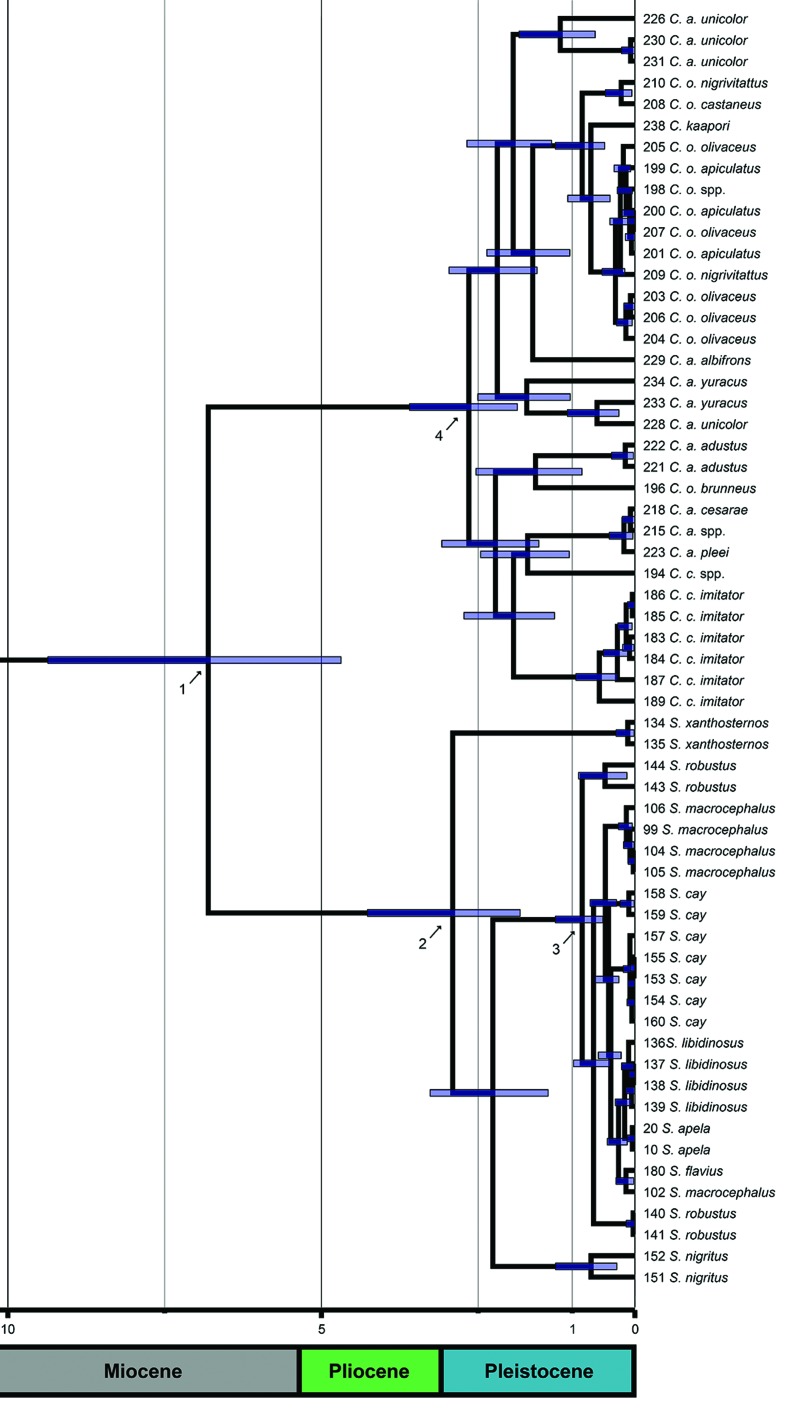
Divergence time tree of capuchins estimated on BEAST 1.8.3. Nodes were calibrated using the age of four Platyrrhini fossils. The blue bars above the nodes indicate the Highest Posterior Density of the estimated ages. Arrows 1, 2, 3 and 4 represent the split of the crown capuchins, the first split of the crown *Sapajus*, a recent split within *Sapajus* (~ 1.0 Ma) and the first split of the crown *Cebus*, respectively. The lower boxes indicate the geological times of the Cenozoic era.

**Figure 4:**
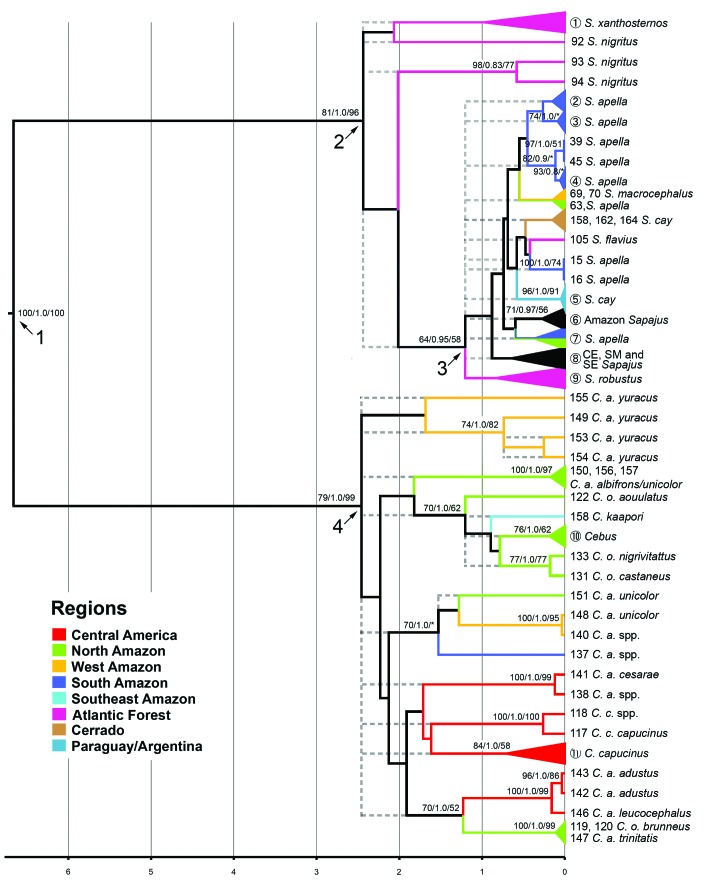
Maximum Clade Credibility (MCC) gene tree estimated on BEAST 1.8.3 from DS2. Circles with Arabic numbers inside represent collapsed samples (see Table S1). The numbers above the nodes are statistical supports provided by, from left to right, Maximum Likelihood bootstrap, Bayesian credibility and Maximum Parsimony bootstrap. The dots show the polytomy in both genera. The meaning of arrows 1, 2, 3 and 4 is described in Figure 3. Except for the nodes indicated by the arrows, only support values greater than 70% are shown.

To reconstruct the ancestral area and the biogeographical history of the main lineages of capuchin monkeys, the R package `BioGeoBEARS’ was used (Matzke, 2013, 2014). A consensus tree with one representative terminal of each main lineage of capuchin monkeys was built and used as “input” to the BioGeoBEARS analyses. The clade represented by the “arrow 3” (Figures 3 and 4) was set as a terminal, except for *S. robustus*.

According to the known geographical distribution for each capuchin species (Silva Jr, 2001; Rylands and Mittermeier, 2013), a pattern of presence/absence for each terminal was coded in a total of eight biogeographical areas, previously proposed by Lynch Alfaro *et al.* (2012) with minor modifications (Figure 2, Table S1): Atlantic Forest (AF) (composed by the North and South Atlantic), Cerrado (CE), Paraguay/Argentina (PA) (representing the Chaco), North Amazon (NM) (North Amazon + Coastal Venezuela), West Amazon (WM), North Andes/Central America (CN), South Amazon (SM) and Southeast Amazon (SE).

All six models present in the `BioGeoBEARS’ package were tested to explain the biogeographical history of the capuchin monkeys: DEC, DEC-J, DIVALIKE, DIVALIKE-J, BAYAREALIKE, BAYAREALIKE-J. The choice of the best model was done checking the lower estimated AIC value.

In order to test whether the observed discrepancies (see Results and Discussion sections below) between our results and that of Lima *et al.* (2017) is due to the difference in the number of biogeographic areas, we ran the BioGeoBEARS program with the four areas proposed by these authors (see Methods Appendix S1).

### Phylogenetic inferences and estimates of divergence times

Phylogenetic reconstructions were estimated based on three different criteria: Maximum Likelihood (ML), Bayesian Inference (BI), and Maximum Parsimony (MP). The RAxMLHPC-AVX 8.2.4 (Stamatakis, 2014) software was used to estimate ML trees, using the models and schemes shown in Table S2. A thousand searches for the most likelihood tree were made, using a random parsimony tree as the starting tree. Node support was provided per 1,000 pseudo replicates of bootstrap.

The MP analyses were carried out in TNT 1.5 beta (Goloboff *et al.*, 2016) software. The New Technology tree search method was used through different search algorithms – RAS, TBR, Tree Drifting, Tree fusing, Ratchet and Sectorial Searches (random and constraint) (Goloboff, 1999). In each search, the best score value had to be found 1,000 times before stop. The support value of the nodes was provided by 1,000 pseudo replications of bootstrap using all the previously used search algorithms. Only bootstrap values above 90% were considered significant.

The BI analyses were carried out by four independent runs in the software BEAST 1.8.3 (Drummond *et al.*, 2012). To estimate divergence times and phylogenetic relationships between different lineages of capuchin monkeys, a non-correlated lognormal relaxed molecular clock model was used to calibrate the tree through a uniform distribution of the crown age of the capuchin monkeys and their previously estimated 95% HPDs (upper value = 9.36; lower value = 4.69). All other priors were set as default.

Four independent MCMC analyses for each dataset were run with 100,000,000 generations being sampled for every 10,000 generations. To check the convergence of the chains and for building a Maximum Clade Credibility (MCC) gene tree, all procedures and softwares described in the previously section about divergence time estimates among capuchin monkeys and other Platyrrhini were used.

## Results

### Sequences and *numts*

A total of 137 new sequences were produced, 72 for the partial Control Region and 65 partial or total sequences of the Cyt *b* gene. All Cyt *b* sequences presented the typical open reading frame for this gene. BLASTn analysis confirmed the mitochondrial characteristics of the Cyt *b* and Control Region sequences, confirming that they are composed of true mtDNA.

### Crown ages and phylogeny of the capuchins

The crown age estimated for capuchins was approximately 6.8 Ma, that is, during the late Miocene (Table 1, Figures 3 and 4). The two databases agreed on a crown age for *Sapajus* ranging between the late Pliocene and early Pleistocene (Table 1), with ages varying from 2.44 Ma (DS2) to 2.91 Ma (DS1). Similarly, the inferences on divergence time indicate that *Cebus* began to diversify in the early Pleistocene, between 2.46 Ma (DS2) and 2.65 Ma (DS1).

**Table 1:** Divergence times for capuchin monkeys and their HPD (Highest Posterior Density) intervals estimated from four different databases. Arrows 1, 2, 3 and 4 can be seen in Figures 3 and 4.

	TimeDS1 [HPD]	TimeDS2 [HPD]
Arrow 1 (Capuchins)	6.8 [4.69 - 9.36]	6.66 [4.69 - 9.04]
Arrow 2 (*Sapajus*)	2.91 [1.83 - 4.27]	2.44 [1.38 - 3.79]
Arrow 3	0.84 [0.51 - 1.27]	1.2 [0.71 - 1.87]
Arrow 4 (*Cebus*)	2.65 [1.88 - 3.59]	2.46 [1.49 - 3.73]

The topologies recovered by the different phylogenetic criteria were broadly congruent (Figure 4). In all cases, monophyly of the capuchins had maximum statistical support, although the statistics were less conclusive for the monophyly of the genera *Cebus* and *Sapajus* (Figure 4). In *Cebus*, the BI and MP analyses confirmed the monophyly of the genus most emphatically, while ML provided an unsatisfactory value (*BS* = 79, Figure 4). In the case of *Sapajus*, monophyly was supported by BI and MP analyses, while ML did not support it (*BS* = 81).

Some well-supported clades were also recovered within each genus, based on the different analyses, although these clades form a polytomy within the genera, impeding the recovery of monophyly or the phylogenetic relationships among the species (Figure 4).

The analyses recovered two clades in *Sapajus*, one formed by two *S. nigritus* samples (93 and 94), which did not group with the other specimen of the same species (92), and the other formed by the *S. cay* samples from the Paraguay/Argentina region (95–98 and 102), which did not group with the samples of the same species from the Brazilian Cerrado (Figure 4).

Despite the wide lack of statistical support for *Sapajus*, some insights are discussed. In all analyses, *S. xanthosternos* and “*S. nigritus*” were the first lineage to diversify in the early robust capuchin evolution. After that, all other robust capuchin lineages are grouped in a polytomy that diverged relatively recently (~ 0.8 to ~ 1.2 Ma) as indicated by arrow 3 in Figures 3 and 4, which has *S. robustus* as the first offshoot. Monophyly and the relationships among lineages in this clade could not be recovery with any kind of support.

Six distinct lineages were recovered for *Cebus* (Figure 4). One of these clades consisted of *C. a. unicolor* from the western Amazon (140 and 148). A second clade was formed by *C. a. albifrons* plus one sample of *C. a. unicolor*, also from the northern Amazon (150, 156 and 157), a third encompassed the subspecies *C. a. cesarae* (138 [*C. a.* spp.] and 141) from the northern Andes, a fourth, the northern Andean *C. a. adustus* (142 and 143) and *C. a. leucocephalus* (146), and a fifth clade included *C. c. capucinus* (117) and *C. capucinus* spp. (118), from Central America. Finally, there is a polytomy involving *C. a. trinitatis* (147) and *C. o. brunneus* (119 and 120).

The position of *C. kaapori* could not be determined. Despite grouping in all analyses with samples of *C. olivaceus* spp., this arrangement was not statistically supported.

### BioGeoBEARS biogeographical history

**Figure 5:**
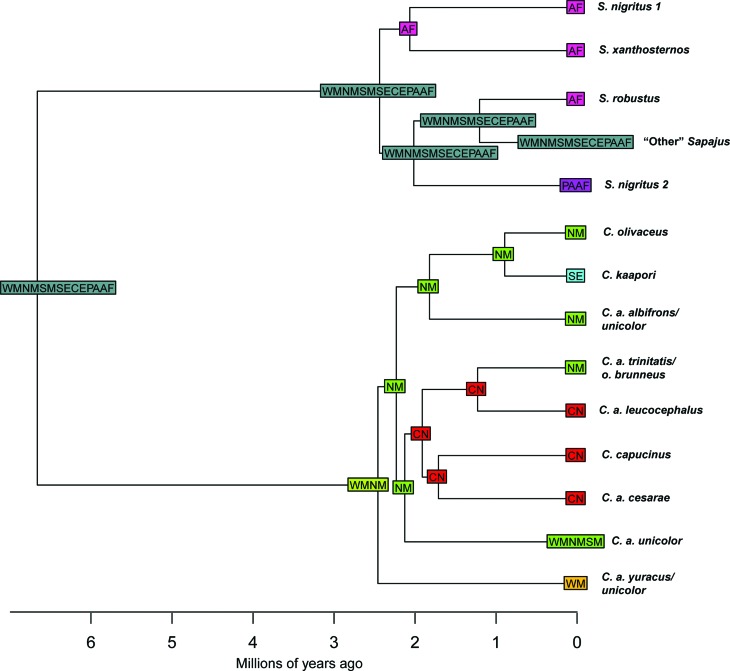
DS2 time consensus tree with the estimates of ancestral areas of the capuchin monkeys made in `BioGeoBEARS’ through the DEC-J model. CN = Central America + North Andes; WM = West Amazon; NM = North Amazon + Coastal Venezuela; SM = South Amazon; SE = Southeast Amazon; CE = Cerrado; PA = Paraguay/Argentina; AF = South and North Atlantic Forest.

According to BioGeoBEARS, the best-fit model explaining the biogeographical history of the capuchin monkeys was DEC+J (Table 2). The model shows that the ancestor of all capuchin monkeys had a wide distribution in South America, from the Atlantic Forest to different regions of the Amazon (Figure 5). The origin of both genera occurred through a sympatric speciation, indicating that *Sapajus* remained within the whole ancestral distribution, while *Cebus* was restricted along the West and North Amazon regions. This means that both gracile and robust capuchins were always present in the Amazon since their lineage split.

**Table 2:** Comparison among the estimated models in `BioGeoBEARS’. For each implemented model in the analyses are shown: the log-likelihood values (LnL), number of parameters (n. params), dispersion, extinction, founder, and values of Akakie’s Information Criteria (AIC and AIC weight).

	LnL	n. params.	dispersion	extinction	founder	AIC	AIC weight
DEC+J	-36.280	3	0.023	10^-12^	0.05	78.56	0.85
DEC	-39.043	2	0.032	10^-12^	0.00	82.09	0.15
BAYAREALIKE+J	-40.251	3	0.020	10^-7^	0.19	86.5	1.00
DIVALIKE+J	-42.341	3	0.038	8.94^-9^	0.03	90.68	0.53
DIVALIKE	-43.463	2	0.045	5.0^-8^	0.00	90.93	0.47
BAYAREALIKE	-49.790	2	0.048	2.91^-1^	0.00	103.6	0.00

The biogeographic history of *Sapajus* was also directed by multiple and independent processes of sympatric speciation, with some lineages arising in the Atlantic Forest or Chaco in the early diversification of the genus, while other populations always remained with a wide distribution along the Cerrado, Atlantic Forest, Amazon and Chaco (Figure 5). Most recently (~ 1.2 Ma, arrow 3, Figure 5), a sympatric speciation has given arise to the *S. robustus* lineage in the Atlantic Forest and to the “other” *Sapajus* lineage, that has a wide distribution in South America.

In the gracile capuchins, vicariance, expansion and founder events drove the diversification of the lineages through time. The initial diversification of *Cebus* occurred by a vicariance process between West and North Amazon. From the North Amazon, different diversification processes occurred reaching different regions. One lineage reached the Central America and Northern Andes by a founder-event in the early diversification of the genus (~ 2 Ma), followed by a recent return to the Northern Amazon. Another lineage remained in the Amazon, reaching recently the Southeast Amazon also by a founder-event, and another one expanded its distribution to the South and West Amazon. The results of the BioGeoBEARS analyses with the four areas of Lima *et al.* (2017) was the same found by Lima *et al.* (2017) (Table S3 and Figure S1).

## Discussion

### The origin of the capuchins

As already suggested by different studies using different kinds of molecular markers, the capuchin monkeys constitute a monophyletic assemblage. However, the estimates of the capuchins origin provided by our data are slightly older than those proposed by Lynch Alfaro *et al.* (2012) and almost 1 Ma older than those found by Lima *et al.* (2017). Probably, the use of different markers is the reason for these differences.

Interestingly, the monophyly of *Cebus* and *Sapajus* could not be significantly recovered by all reconstruction analyses; while the BI and MP analyses recovered it, the ML analyzes did not. This can be explained by possible past introgression across these two lineages along their evolution, as shown in the mitochondrial markers used (Nascimento *et al.*, 2015; Ruiz-García *et al.*, 2016; Lima *et al.*, 2017). As many other studies using multiple nuclear molecular markers have confirmed the monophyly of the gracile and robust capuchins, we do not consider that the lack of support in this study represents a real case of paraphyly for these genera (Perelman *et al.*, 2011; Martins-Junior *et al.*, 2015).

The timing of the origin of the capuchins during the late Miocene, around 6.8 Ma, is similar to that of other platyrrhines, such as the subgenus *Saguinus sensu* Garbino and Martins-Junior (2018) (Perelman *et al.*, 2011; Buckner *et al.*, 2015; Rylands *et al.*, 2016), *Ateles* (Morales-Jimenez *et al.*, 2015) and *Alouatta* (Cortés-Ortiz *et al.*, 2003; Nascimento *et al.*, 2005; Perelman *et al.*, 2011). The origin of the two genera is more consistent with the relatively recent diversification of some lineages, such as *Saimiri* (Lynch Alfaro *et al.*, 2015; Mercês *et al.*, 2015), *Callithrix* (Schneider *et al.*, 2012), *Mico* (Perelman *et al.*, 2011; Schneider *et al.*, 2012), *Brachyteles* and *Lagothrix* (Perelman *et al.*, 2011; Di Fiori *et al.*, 2015) and *Callicebus* and *Cheracebus* (Byrne *et al.*, 2016).

### Phylogenetic and taxonomic implications in *Cebus* and *Sapajus*

The phylogenetic analyses were unable to clarify the relationships among the different species (Figure 4). This strongly indicates that the evolution within *Cebus* and *Sapajus* probably resulted from an explosive process of diversification and dispersal between 2 and 3 Ma, as pointed out by several studies with this group (Casado *et al.*, 2010; Boubli *et al.*, 2012; Lynch Alfaro *et al.*, 2012; Martins-Junior *et al.*, 2015; Lima *et al.*, 2017).

The evolution of the capuchins appears to have been far more complex than that of other platyrrhines, given that, in most cases, the Cyt *b* and Control Region have been used successfully to confirm the monophyly of the species of several other genera (Bonvicino *et al.*, 2001, 2003; Cortés-Ortiz *et al.*, 2003; Nascimento *et al.*, 2005; Babb *et al.*, 2011; de Mello Martins *et al.*, 2011; Botero *et al.*, 2015; Morales-Jimenez *et al.*, 2015; Lynch Alfaro *et al.*, 2015). Even so, unresolved polytomies have also been found in most cases. Similar low levels of genetic differentiation have also been found in several vertebrate taxa distributed in both the Atlantic and Amazon forests (Costa, 2003; Santos *et al.*, 2007; Cabanne *et al.*, 2007; Casado *et al.*, 2010; Martins-Junior *et al.*, 2015).

An additional factor that may have enhanced the complexity of this process is the hybridization of lineages, both recent and ancient. The existence of extensive zones of contact between most species (Figure 1) strongly indicates that hybridization may have been frequent during the evolution of the lineages during the Pleistocene, a process that may be ongoing (Santos *et al.*, 1987; Coimbra-Filho *et al.*, 1991-1992; Silva Jr, 2001; Lynch Alfaro *et al.*, 2012).

An important result of this study is that monophyly was not obtained for any of the species of robust capuchin monkeys. This situation is similar to the ones found by Casado *et al.* (2010), Ruiz-García *et al.* (2012) and Lima *et al.* (2017), also using mitochondrial genes. Lima *et al.* (2017), however, found support for the monophyly of *S. nigritus* and *S. xanthosternos*. Martins-Junior *et al.* (2015) also found a polytomic pattern in nuclear markers, indicating an explosive process of diversification during evolutionary history (Lynch Alfaro *et al.*, 2012; Martins-Junior *et al.*, 2015).

Our estimates were not able to establish the position of *S. flavius* within the clade, as indicated by arrow 3 in Figure 4. From the different phylogenetic criteria, its position varies in the topology. Even though *S. flavius* presents different characters that make it a full species (see Oliveira and Langguth, 2006), new studies must be made with more molecular markers to test its phylogenetic position.

The results of the current study agree with the findings of Casado *et al.* (2010), in relation to the formation of a monophyletic group composed by the *S. cay* specimens from Paraguay/Argentina, which are quite distinct from specimens from the Brazilian Cerrado, but which do not coalesce into a clade, suggesting that the taxon needs more attention. In addition to *S. cay*, a number of *S. apella* lineages were recovered without statistical support, and a very recent origin (~ 500 Ka), but without forming a single monophyletic group for the species. Ruiz-García *et al.* (2012) were also unable to separate the different *S. apella* subspecies into distinct clades. The authors show a strict relationship between *S. cay* (samples from Paraguay and Mato Grosso in Brazil) and *S. macrocephalus* suggesting the former as a subspecies of *S. apella*. They also found that the samples from Yungas in Bolivia-Argentina, classified by Silva Jr (2001) as *S. cay*, are more similar with samples of *S. macrocephalus* than the other putative *S. cay*. Casado *et al.* (2010), using Cyt *b*, found only a subtle genetic difference between the *S. cay* and *S. apella*, with a polytomy between these lineages. More recently, Lima *et al.* (2017) could not recover the monophyly of *S. apella*, *S. macrocephalus* and *S. cay*. In their work, samples of *S. cay* from the Cerrado and Amazonia biomes in Mato Grosso and material from Paraguay are genetically very similar with samples of *S. apella* from southern Amazon in Mato Grosso and Rondonia in Brazil.

A very similar pattern was observed between *S. apella* and *S. macrocephalus*. Our results show no genetic differences between these two lineages, as have been shown in previous studies (Ruiz-García *et al.*, 2012; Lynch Alfaro *et al.*, 2012; Lima *et al.*, 2017). Groves (2001) proposes that morphological differences between these two species are very small, mainly when *S. macrocephalus* is compared to *S. apella fatuellus*, indicating that *macrocephalus* may be a subspecies of *S. apella*. Silva Jr (2001) suggested that *S. macrocephalus* was a valid species, but mentioned several morphological similarities between it and *S. apella*. On the other hand, Ruiz-García *et al.* (2012), using genetic population and phylogenetic analysis, found that, even though *S. macrocephalus* and *S. apella* form different populations, the differences between them are too low to be split in two different species. This absence of genetic differences was also highlighted by Lima *et al.* (2017).

From a more conservative perspective, with the exception of *S. xanthosternos* and *S. nigritus*, most recognized species appear to have diverged within the past million years (arrow 3, Figure 4). This is a relatively short period of time for the establishment of synapomorphies among the distinct taxonomic entities (Casado *et al.*, 2010), especially as the historical diversification process would have occurred in association with hybridization events. One consequence of this would be the enormous phenotypic diversity found in the robust capuchins (inter and intraspecifically), which may reflect the lack of an adequate time scale for the establishment of distinct morphological lineages. This, together with the findings from the two mitochondrial markers analyzed here, precludes the recognition of the different *Sapajus* species.

Even though Lima *et al.* (2017) used three mitochondrial markers and found monophyly for *S. nigritus* and *S. xanthosternos*, they did not use other phylogenetic methods to recover the monophyly of these species. In this context, the current evidence indicates that the diversity of robust capuchin species seems to be lower than the proposed by Silva Jr (2001). Here, considering all the morphological, biogeographical and genetic evidences discussed, we agree with the previous proposal by Ruiz-García *et al.* (2012), that *S. cay* and *S. macrocephalus* are junior synonyms of *S. apella*. Even with a biogeographical difference between *S. cay* and *S. apella* – the former occurring preferentially outside the Amazon – this study, as well as previous ones (Ruiz-Garcia *et al.*, 2012; Lima *et al.*, 2017), show that the two taxa are not differentiated with respect to traditional molecular markers. In the case of *S. macrocephalus*, in addition to genetic and morphological evidence, both it and *S. apella* occur continuously in the Amazon and the geographical boundaries between these two lineages are not well defined (Groves, 2001; Silva Jr, 2001). New studies with more molecular markers from nuclear and mitochondria or a genomic approach will certainly clarify this question. Morphologically, more studies involving geographic variation in pelage may show that the diagnostic pelage features of the traditionally recognized species correspond to a clinal variation.

It is interesting to note that in *Cebus*, a number of lineages did form well-supported clades (Figure 4), even though the evolutionary relationships among them were not well established. The preliminary analysis of the diversity of *Cebu,* done by Boubli *et al.* (2012) based on Cyt *b* and Control Region and using a pure BI analysis pointed to the existence of six groups and a total of 12 species, challenging the accepted arrangement (*C. albifrons*, *C. olivaceus*, *C. kaapori* and *C. capucinus*). This proposal was accepted partially by Rylands and Mittermeier (2013), who proposed 14 *Cebus* species.

However, none of the groups proposed by Boubli *et al.* (2012) were recovered with adequate statistical support in the current study, although some findings were concordant. For example, the specimens of *C. a. albifrons* formed a monophyletic group, while *C. a. leucocephalus* and *C. a. adustus* coalesced into a monophyletic group, indicating that these two forms do, in fact, represent a single subspecies. Similarly, the samples of *C. o. brunneus* and *C. a. trinitatis* also formed a monophyletic, indicating that they form a single taxonomic unit.

Some authors suggest that *C. a. trinitatis*, from Trinidad Tobago Island, could have originated from an ancestral population of *C. albifrons* from the Venezuelan Andes (Boubli *et al.*, 2012). Others suggest that these animals were introduced in the island by humans from Venezuelan populations of *C. olivaceus* (Long, 2003). However, the clear morphological distinctions between *C. a. trinitatis* and *C. o. brunneus* highlighted when specimens from museums or from the wild are compared, make this grouping unexpected. New studies with more samples are necessary to clarify this question.

The *C. a. cesarae* clade was also supported statistically. The position of *C. kaapori* is still controversial, with some authors proposing it as a subspecies of *C. olivaceus* (Rylands *et al.*, 2000), and others considering it a full species (Groves, 2001; Silva Jr, 2001). Even though our analyses agree with Lima *et al.* (2017), grouping *C. kaapori* with some *C. olivaceus* lineages, this arrangement had no statistical support (Figure 4).

Overall, the lack of any clear monophyly in the different species, together with clear polytomy of the different *Cebus* lineages, restricts any conclusive interpretation of the different arrangements proposed for the *Cebus* species. In the absence of well-supported evidence, we suggest the continued use of the conventional taxonomic arrangement currently used by most authors (*C. albifrons*, *C. olivaceus*, *C. kaapori* and *C. capucinus*) instead of the proposal by Boubli *et al.* (2012), at least until additional mitochondrial, and principally, nuclear markers are analyzed.

### Not a recent but an old sympatric event explains the current sympatry between gracile and capuchin monkeys

The most important result obtained in the current research is that *Cebus* and *Sapajus* arose at ~ 6.8 Ma from a sympatric event, with both genera occurring in the Amazon since their origin (Figure 5). Furthermore, both genera have always been present in the Amazon throughout their biogeographic history. It means that the current sympatry observed between gracile and robust capuchins in the Amazon is explained by an ancestral distribution of these two lineages, supporting the Sympatric Evolution (SEV) hypothesis. It is an important finding because the currently accepted hypothesis based on previous studies is the Reinvasion of the Amazon (Lynch Alfaro *et al.*, 2012; Lima *et al.*, 2017), which states that only recently have the robust capuchins colonized the Amazon basin. In none of the previous studies a scenario of sympatric evolution was considered.

Lima *et al.* (2017) found that the ancestral capuchins had a wide distribution throughout South America, from the Amazon to the Atlantic Forest. These authors also suggest that the formation of the Cerrado (4 – 8 Ma) was the geographical barrier responsible for the vicariant origin of *Cebus* and *Sapajus*, with the former restricted to the Amazon and the latter to the Atlantic Forest. Our results agree with this wide distribution of ancestral capuchins. However, even if the Cerrado has had an important role in the origin of these two genera, it was not a geographical barrier for them, since they arose by a sympatric process in the Amazon, with *Sapajus* widespread across all South American regions and *Cebus* restricted to North and West Amazon (Figure 5).

In the case of the historical biogeography of *Sapajus*, strong discrepancies were found here when compared to the results of Lima *et al.* (2017). The latter authors found that *Sapajus* was restricted to the Atlantic Forest along most of its evolutionary history and only recently (at c. 500 Ka) expanded their distribution to the Cerrado, Chaco and Amazon regions. In contrast, our analyses do not support this proposal, but show that the robust capuchins always had a wide distribution across these different biomes, and that the origin of the distinct lineages occurred by multiple and independent sympatric events (Figure 5).

In the recovered topologies, the grouping of *S. apella* samples from both banks of the Amazon rivers (Figure 4) suggest that these rivers were and are not geographical barriers for these primates, contrary what has been observed for other Platyrrhini groups (Vallinoto *et al.*, 2006; Couette, 2007; Boubli *et al.*, 2015; Lynch Alfaro *et al.*, 2015; Mercês *et al.*, 2015). This result agrees with the finds of Lima *et al.* (2017). This can be explained by the wide ancestral and continued distribution of *Sapajus* across different regions of the Amazon (Figure 5). Furthermore, it suggests that throughout the evolution of the genus there was gene flow between robust capuchin populations from different river banks.

It is important to note that these discrepancies between our results and those found by Lima *et al.* (2017) are explained by the use of different geographical areas (Figure 5, Figure S1 and Table S3). While the analysis in BioGeoBEARS with the eight areas from Lynch Alfaro *et al.* (2012) corroborate the Sympatric Evolution Hypothesis, the analysis with four areas proposed by Lima *et al.* (2017) support the Reinvasion of the Amazon Hypothesis. Although this weakens our result of a sympatric origin for *Cebus* and *Sapajus*, it also shows that the historical biogeography of the capuchin monkeys is still in debate, as the data from both the present study and the one by Lima *et al.* (2017) do not satisfactorily solve this question. This also shows that the scientific community should be careful in defining the biogeographic areas for the BioGeoBEARS analyses, especially if the data is not phylogenetically strong.

Regarding *Cebus*, the analyses support the proposal of Lynch Alfaro *et al.* (2012) and Lima *et al.* (2017) for an Amazon origin of the gracile capuchins (Figures 4 and 5). In fact, it appears that *Cebus* has experienced different kinds of diversification processes along its evolutionary history. While the diversification of *Sapajus* was driven by sympatric speciation, after an initial vicariance event, *Cebus* experienced founder and range expansion events. In their early diversification (~ 2 Ma), the gracile capuchins crossed the Amazon river southward into the South Amazon, and crossed the Andes northwards reaching Central America. In contrast, it seems that the Tocantins river was a strong barrier for this group, since *Cebus* reached the Southeast Amazon basin only recently (Figure 5). Our results agree with those found by Lima *et al.* (2017) about the incursion into Central America by gracile capuchin monkeys at ~ 2 Ma, after the complete elevation of the Andes and the closure of the Panama Isthmus (Hoorn *et al.*, 2010).

Three important questions remain to be answered: Which of the biogeographical hypotheses for capuchins evolution is more plausible: Sympatric Evolution (SE) or Reinvasion of the Amazon (ROA)? When and which putative routes were used by gracile capuchins to cross the Andes Cordillera? Why did *Sapajus* even come to exist in the Amazon, since by its origin it could not cross the Andes? Certainly, new studies with samples from these key regions and using more molecular markers, mainly NGS data, will reveal which is the most likely phylogeographical scenario.

## Supplementary material

The following online material is available for this article:

Figure S1 - DS2 time consensus tree with the estimates of ancestral areas of the capuchin monkeys considering the four areas proposed by Lima *et al.* (2017).

Table S1 - List of species with their respective samples, codes, ID.

Table S2 - Characteristics of the datasets.

Table S3 - Comparison among the estimated models in `BioGeoBEARS’ considering the four areas.

Methods Appendix S1 - Methods used in `BioGeoBEARS’ with the four areas from Lima *et al.* (2017).
