# Supplementary material for: Phylogenetic relationships among Capuchin (Cebidae, Platyrrhini) lineages: An old event of sympatry explains the current distribution of Cebus and Sapajus
Source: Genet Mol Biol. 2018 Jul-Sep;41(3):699–712. doi: 10.1590/1678-4685-GMB-2017-0012 (PMC6136366; doi:10.1590/1678-4685-GMB-2017-0012)
Supplement: Supplementary file 1 [file 1415-4757-GMB-41-03-2017-0012-20180717-suppl2.pdf]

Supplementary Material to "Phylogenetic relationships among Capuchin (Cebidae, Platyrrhini) lineages: An old event of sympatry explains the current distribution of *Cebus* and *Sapajus*"

Table S1 – List of species with their respective samples, codes, ID, source, coordinates, geographical location, biogeographic zone, GenBank access numbers, and datasets. The numbers within the circles in the datasets columns refer to the collapsed clades in the tree constructed for the dataset2 (DS2) in Figure 4.

**Species**	**Code**	**ID**	**Source**	**Lat.**	**Long.**	**Locality**	**Region**	**GenBank Cyt *b***	**GenBank D-loop**	**DS1**	**DS2**
Sapajus apella	1 *S. apella*	Caa02	PGLab	-3.90	-49.52	Tucuruí HPP - TR, PA, BR	Southeast Amazon	MF472527	MF472455	----	X⑥
	2 *S. apella*	Caa04	PGLab	-3.90	-49.52	Tucuruí HPP - TR, PA, BR	Southeast Amazon	MF472528	MF472456	----	X⑥
	3 *S. apella*	Caa25	PGLab	-3.90	-49.52	Tucuruí HPP - TR, PA, BR	Southeast Amazon	MF472529	MF472457	----	X⑧
	4 *S. apella*	Caa361	PGLab	-3.90	-49.52	Tucuruí HPP - TR, PA, BR	Southeast Amazon	-----------	MF472458	----	X⑧
	5 *S. apella*	Caa1099	PGLab	-3.85	-49.78	Tucuruí HPP - TR, PA, BR	South Amazon	MF472530	MF472463	X	X⑧
	6 *S. apella*	Caa335	PGLab	-3.85	-49.78	Tucuruí HPP - TR, PA, BR	South Amazon	MF472531	MF472459	----	X⑧
	7 *S. apella*	Caa339	PGLab	-3.85	-49.78	Tucuruí HPP - TR, PA, BR	South Amazon	MF472532	MF472460	----	X⑧
	8 *S. apella*	Caa449	PGLab	-3.85	-49.78	Tucuruí HPP - TR, PA, BR	South Amazon	MF472533	MF472461	----	X⑧
	9 *S. apella*	Caa993	PGLab	-3.85	-49.78	Tucuruí HPP - TR, PA, BR	South Amazon	MF472534	MF472462	X	X⑧

Species	Code	ID	Source	Lat.	Long.	Locality	Region	GenBank Cyt *b*	GenBank D-loop	DS1	DS2
	10 *S. apella*	Caa1684	PGLab	-3.85	-49.78	Tucuruí HPP - TR, PA, BR	South Amazon	-----------	MF472465	----	X⑧
	11 *S. apella*	Caa1311	PGLab	-3.85	-49.78	Tucuruí HPP - TR, PA, BR	South Amazon	-----------	MF472464	----	X⑧
	12 *S. apella*	Caa60	PGLab	-3.33	-52.10	Altamira, XR, PA, BR	South Amazon	MF472535	MF472467	----	X⑦
	13 *S. apella*	Caa82	PGLab	-3.19	-52.21	Altamira, XR, PA, BR	South Amazon	MF472536	MF472468	----	X⑦
	14 *S. apella*	Caa46	PGLab	-3.19	-52.21	Altamira, XR, PA, BR	South Amazon	MF472537	MF472466	----	X⑦
	15 *S. apella*	Parna 067	PGLab	-4.66	-56.75	Itaituba, PNA, TPR, PA, BR	South Amazon	MF472538	MF472469	----	X
	16 *S. apella*	Parna 068	PGLab	-4.66	-56.75	Itaituba, PNA, TPR, PA, BR	South Amazon	MF472539	MF472470	----	X
	17 *S. apella*	Caa2212	PGLab	-8.74	-63.44	Samuel HPP - JR, RO, BR	South Amazon	MF472540	MF472471	----	X②
	18 *S. apella*	Caa2251	PGLab	-8.74	-63.44	Samuel HPP - JR, RO, BR	South Amazon	MF472541	MF472472	----	X②
	19 *S. apella*	Caa2260	PGLab	-8.74	-63.44	Samuel HPP - JR, RO, BR	South Amazon	MF472542	MF472473	----	X②
	20 *S. apella*	Caa2266	PGLab	-8.74	-63.44	Samuel HPP - JR, RO, BR	South Amazon	MF472543	MF472474	----	X②
	21 *S. apella*	Caa2267	PGLab	-8.74	-63.44	Samuel HPP - JR, RO, BR	South Amazon	MF472544	MF472475	----	X②
	22 *S. apella*	Caa2268	PGLab	-8.74	-63.44	Samuel HPP - JR, RO, BR	South Amazon	MF472545	MF472476	----	X②

Species	Code	ID	Source	Lat.	Long.	Locality	Region	GenBank Cyt *b*	GenBank D-loop	DS1	DS2
	23 *S. apella*	Caa2288	PGLab	-8.74	-63.44	Samuel HPP - JR, RO, BR	South Amazon	MF472546	MF472477	----	X②
	24 *S. apella*	Caa2303	PGLab	-8.74	-63.44	Samuel HPP - JR, RO, BR	South Amazon	MF472547	MF472478	----	X②
	25 *S. apella*	Caa2313	PGLab	-8.74	-63.44	Samuel HPP - JR, RO, BR	South Amazon	MF472548	MF472479	----	X②
	26 *S. apella*	Caa2348	PGLab	-8.74	-63.44	Samuel HPP - JR, RO, BR	South Amazon	MF472549	MF472480	----	X②
	27 *S. apella*	Caa2364	PGLab	-8.74	-63.44	Samuel HPP - JR, RO, BR	South Amazon	MF472550	MF472481	----	X②
	28 *S. apella*	Caa2365	PGLab	-8.74	-63.44	Samuel HPP - JR, RO, BR	South Amazon	MF472551	MF472482	----	X②
	29 *S. apella*	Caa2385	PGLab	-8.74	-63.44	Samuel HPP - JR, RO, BR	South Amazon	MF472552	MF472483	----	X②
	30 *S. apella*	Caa2447	PGLab	-8.74	-63.44	Samuel HPP - JR, RO, BR	South Amazon	MF472553	MF472484	----	X②
	31 *S. apella*	Caa4463	PGLab	-8.74	-63.44	Samuel HPP - JR, RO, BR	South Amazon	MF472554	MF472485	----	X②
	32 *S. apella*	Caa4471	PGLab	-8.74	-63.44	Samuel HPP - JR, RO, BR	South Amazon	MF472555	MF472486	----	X②
	33 *S. apella*	Caa4600	PGLab	-8.74	-63.44	Samuel HPP - JR, RO, BR	South Amazon	MF472556	MF472487	----	X②
	34 *S. apella*	Caa4621	PGLab	-8.74	-63.44	Samuel HPP - JR, RO, BR	South Amazon	MF472557	MF472488	----	X②

**Species**	**Code**	**ID**	**Source**	**Lat.**	**Long.**	**Locality**	**Region**	**GenBank Cyt *b***	**GenBank D-loop**	**DS1**	**DS2**
	35 *S. apella*	Caa4511	PGLab	-8.76	-63.48	Samuel HPP - JR, RO, BR	South Amazon	MF472558	MF472489	----	X②
	36 *S. apella*	Caa4530	PGLab	-8.76	-63.48	Samuel HPP - JR, RO, BR	South Amazon	MF472559	MF472490	----	X②
	37 *S. apella*	Caa4330	PGLab	-8.74	-63.44	Samuel HPP - JR, RO, BR	South Amazon	MF472560	MF472491	----	X④
	38 *S. apella*	Caa4331	PGLab	-8.74	-63.44	Samuel HPP - JR, RO, BR	South Amazon	MF472561	MF472492	----	X④
	39 *S. apella*	Caa4608	PGLab	-8.74	-63.44	Samuel HPP - JR, RO, BR	South Amazon	MF472562	MF472493	----	X
	40 *S. apella*	Caa2367	PGLab	-8.76	-63.48	Samuel HPP - JR, RO, BR	South Amazon	MF472563	MF472494	----	X④
	41 *S. apella*	Caa2368	PGLab	-8.76	-63.48	Samuel HPP - JR, RO, BR	South Amazon	MF472564	MF472495	----	X④
	42 *S. apella*	Caa2373	PGLab	-8.76	-63.48	Samuel HPP - JR, RO, BR	South Amazon	MF472565	MF472496	----	X④
	43 *S. apella*	Caa2403	PGLab	-8.76	-63.48	Samuel HPP - JR, RO, BR	South Amazon	MF472566	MF472497	----	X④
	44 *S. apella*	Caa4341	PGLab	-8.76	-63.48	Samuel HPP - JR, RO, BR	South Amazon	MF472567	MF472498	----	X④
	45 *S. apella*	Caa4342	PGLab	-8.76	-63.48	Samuel HPP - JR, RO, BR	South Amazon	MF472568	MF472499	----	X
	46 *S. apella*	Caa4626	PGLab	-8.76	-63.48	Samuel HPP - JR, RO, BR	South Amazon	MF472569	MF472500	----	X④
	47 *S. apella*	Caa4680	PGLab	-8.76	-63.48	Samuel HPP - JR, RO, BR	South Amazon	MF472570	MF472501	----	X④

Species	Code	ID	Source	Lat.	Long.	Locality	Region	GenBank Cyt *b*	GenBank D-loop	DS1	DS2
	48 *S. apella*	Caa4554	PGLab	-8.74	-63.44	Samuel HPP - JR, RO, BR	South Amazon	MF472571	MF472502	----	X③
	49 *S. apella*	Caa4555	PGLab	-8.74	-63.44	Samuel HPP - JR, RO, BR	South Amazon	MF472572	MF472503	----	X③
	50 *S. apella*	Caa4556	PGLab	-8.74	-63.44	Samuel HPP - JR, RO, BR	South Amazon	MF472573	MF472504	----	X③
	51 *S. apella*	Caa2350	PGLab	-8.76	-63.48	Samuel HPP - JR, RO, BR	South Amazon	MF472574	MF472505	----	X③
	52 *S. apella*	Caa2352	PGLab	-8.76	-63.48	Samuel HPP - JR, RO, BR	South Amazon	MF472575	MF472506	----	X③
	53 *S. apella*	Caa2359	PGLab	-8.76	-63.48	Samuel HPP - JR, RO, BR	South Amazon	MF472576	MF472507	----	X③
	54 *S. apella*	Caa2379	PGLab	-8.76	-63.48	Samuel HPP - JR, RO, BR	South Amazon	MF472577	MF472508	----	X③
	55 *S. apella*	Caa2446	PGLab	-8.76	-63.48	Samuel HPP - JR, RO, BR	South Amazon	MF472578	MF472509	----	X③
	56 *S. apella*	Caa4343	PGLab	-8.76	-63.48	Samuel HPP - JR, RO, BR	South Amazon	MF472579	MF472510	----	X③
	57 *S. apella*	Caa4690	PGLab	-8.76	-63.48	Samuel HPP - JR, RO, BR	South Amazon	MF472580	MF472511	----	X③
	58 *S. apella*	Caa2074	PGLab	-0.65	-52.54	SAJ HPP, JAR, PA, BR	North Amazon	MF472581	MF472512	----	X⑦
	59 *S. apella*	Caa2079	PGLab	-0.65	-52.54	SAJ HPP, JAR, PA, BR	North Amazon	MF472582	MF472513	----	X⑦

**Species**	**Code**	**ID**	**Source**	**Lat.**	**Long.**	**Locality**	**Region**	**GenBank Cyt *b***	**GenBank D-loop**	**DS1**	**DS2**
	60 *S. apella*	Caa2517	PGLab	-1.89	-59.62	Balbina HPP, UR, AM, BR	North Amazon	-----------	MF472514	----	X⑥
	61 *S. apella*	Caa3185	PGLab	-1.10	-57.04	CP HPP, TRR, PA, BR	North Amazon	-----------	MF472515	----	X⑥
	62 *S. apella*	Caa3050	PGLab	-1.10	-57.04	CP HPP, TRR, PA, BR	North Amazon	-----------	MF472516	----	X⑥
	63 *S. apella*	Caa3070	PGLab	-1.10	-57.04	CP HPP, TRR, PA, BR	North Amazon	-----------	MF472517	----	X
	64 *S. apella*	296634	USNM	1.35	-58.83	Kanashen, GUY	North Amazon	JN409297	-----------	----	X⑥
	65 *S. apella*	547902	USNM	-2.70	-59.65	Manaus, AM, BR	North Amazon	JN409329	-----------	----	X⑦
S. macrocephalus	66 *S. macrocephalus*	JPB 80	INPA	-0.48	-64.41	Rio Negro, AM, BR	North Amazon	JQ317665	JQ317619	----	X⑥
	67 *S. macrocephalus*	241394	USNM	4.15	-73.63	Villavicencio, COL	West Amazon	JN409324	-----------	----	X⑥
	68 *S. macrocephalus*	397979	USNM	3.72	-73.48	San Martin, Meta, COL	West Amazon	JN409296	-----------	X	----
	69 *S. macrocephalus*	518262	USNM	-3.45	-68.80	Sao Paulo de Olivenca, AM, BR	North Amazon	JN409325	-----------	----	X
	70 *S. macrocephalus*	268240	AMNH	-7.53	-74.97	Galvez River, San J., Loreto, PER	West Amazon	JN409326	-----------	----	X
	71 *S. macrocephalus*	12294	LSUMZ	-10.00	-71.02	Rio Curanja, Balta, Loreto, PER	West Amazon	JN409327	-----------	X	----

**Species**	**Code**	**ID**	**Source**	**Lat.**	**Long.**	**Locality**	**Region**	**GenBank Cyt *b***	**GenBank D-loop**	**DS1**	**DS2**
	72 *S. macrocephalus*	CRB1806	GenBank	-0.96	-62.92	Barcelos, AM, BR	North Amazon	FJ529103	-----------	X	X⑥
	73 *S. macrocephalus*	CRB2632	GenBank	-0.96	-62.92	Barcelos, AM, BR	North Amazon	FJ529104	-----------	X	----
	74 *S. macrocephalus*	CRB2858	GenBank	-0.96	-62.92	Barcelos, AM, BR	North Amazon	FJ529102	-----------	X	----
S. xanthosternos	75 *S. xanthosternos*	LMM37	UESC	-15.18	-39.11	REBIO UNA, BA, BR	North Atlantic	-----------	JQ412900	----	X①
	76 *S. xanthosternos*	LMM35	UESC	-15.18	-39.11	REBIO UNA, BA, BR	North Atlantic	-----------	JQ412898	----	X①
	77 *S. xanthosternos*	LMM34	UESC	-15.18	-39.11	REBIO UNA, BA, BR	North Atlantic	-----------	JQ412897	----	X①
	78 *S. xanthosternos*	LMM33	UESC	-15.18	-39.11	REBIO UNA, BA, BR	North Atlantic	-----------	JQ412896	----	X①
	79 *S. xanthosternos*	518303	USNM	-15.87	-39.12	Belmonte, BA BR	North Atlantic	JN409330	-----------	----	X⑨
	80 *S. xanthosternos*	FJ460174	GenBank	-15.14	-39.16	REBIO UNA, BA, BR	North Atlantic	FJ460174	-----------	X	X①
	81 *S. xanthosternos*	13190	GenBank	-15.18	-39.11	REBIO UNA, BA, BR	North Atlantic	FJ529105	-----------	X	X①
S. libidinosus	82 *S. libidinosus*	slib20	CETAS[2]	-17.86	-49.55	UNK	Cerrado	MF472583	MF472520	X	X⑧
	83 *S. libidinosus*	slib25	CETAS[1]	-17.86	-49.55	UNK	Cerrado	MF472584	MF472521	X	X⑧
	84 *S. libidinosus*	27344	LACM	-15.68	-48.20	Brasilandia, GO, BR	Cerrado	JN409299	-----------	X	X⑧

**Species**	**Code**	**ID**	**Source**	**Lat.**	**Long.**	**Locality**	**Region**	**GenBank Cyt *b***	**GenBank D-loop**	**DS1**	**DS2**
	85 *S. libidinosus*	518369	USNM	-17.22	-46.87	Paracatu, MG, BR	Cerrado	JN409300	-----------	X	----
S. robustus	86 *S. robustus*	srob29	CETAS[1]	-17.47	-41.28	UNK	North Atlantic	MF472585	MF472522	X	X⑨
	87 *S. robustus*	srob39	CETAS[1]	-17.47	-41.28	UNK	North Atlantic	MF472586	MF472523	X	X⑨
	88 *S. robustus*	518434	USNM	−17.8	−41.5	Teofilo Otoni, MG, BR	North Atlantic	JQ317672	JQ317618	----	X⑨
	89 *S. robustus*	518327	USNM	-18.58	-39.75	Conceicao da Barra, ES, BR	North Atlantic	JN409301	-----------	X	X⑨
	90 *S. robustus*	518429	USNM	-17.85	-41.50	Teofilo Otoni, MG, BR	North Atlantic	JN409331	-----------	X	X⑨
	91 *S. robustus*	518311	USNM	-21.23	-41.20	Sao Domingos, ES, BR	North Atlantic	JN409302	-----------	----	X⑨
S. nigritus	92 *S. nigritus*	518478	USNM	-23	-44.30	Angra dos Reis, RJ, BR	South Atlantic	JN409333	JQ317617	----	X
	93 *S. nigritus*	518524	USNM	-20.27	-50.23	Fernandopolis, SP, BR	South Atlantic	JN409304	-----------	X	X
	94 *S. nigritus*	GRU3	U. Mon.	-25.70	-54.44	Iguazu Falls, ARG	Paraguay/Argentina	JN409334	-----------	X	X
S. cay	95 *S. cay*	CAP1832	CENP	-26.61	-57.57	UNK	Paraguay/Argentina	MF472587	MF472524	X	X⑤
	96 *S. cay*	CAP1871	CENP	-26.61	-57.57	UNK	Paraguay/Argentina	MF472588	MF472525	X	X⑤
	97 *S. cay*	124696	UMMZ	-26.88	-56.88	Misiones, PAR	Paraguay/Argentina	JN409298	-----------	X	X⑤

**Species**	**Code**	**ID**	**Source**	**Lat.**	**Long.**	**Locality**	**Region**	**GenBank Cyt *b***	**GenBank D-loop**	**DS1**	**DS2**
	98 *S. cay*	CAP76	GenBank	-26.50	-57.73	Santa Catalina, PAR	Paraguay/Argentina	FJ529076	-----------	X	X⑤
	99 *S. cay*	CA562	GenBank	-15.46	-55.75	Rio Quilombo, MTG, BR	Cerrado	FJ529053	-----------	X	X
	100 *S. cay*	CA549	GenBank	-15.46	-55.75	Rio Quilombo, MTG, BR	Cerrado	FJ529046	-----------	X	----
	101 *S. cay*	CAP475	GenBank	-26.50	-57.73	Santa Catalina, PAR	Paraguay/Argentina	FJ529101	-----------	X	----
	102 *S. cay*	CAP14	GenBank	-26.50	-57.73	Santa Catalina, PAR	Paraguay/Argentina	FJ529071	-----------	----	X⑤
	103 *S. cay*	CA865	GenBank	-14.86	-55.81	Rio Manso, MTG, BR	Cerrado	FJ529068	-----------	----	X
	104 *S. cay*	CA645	GenBank	-14.86	-55.81	Rio Manso, MTG, BR	Cerrado	FJ529066	-----------	----	X
S. flavius	105 *S. flavius*	sfla15	CETAS[2]	-8.61	-35.41	UNK	North Atlantic	MF472589	MF472526	X	X
Cebus capucinus limitaneus	106 *C. c. limitaneus*	65-105	Connor	15.26	-83.78	Puerto Lempira, HON	Central America	-----------	JQ317620	----	X⑪
C. c. imitator	107 *C. c. imitator*	CC56	UCR	10.35	-85.35	Palo Verde, E. MINAE, CR	Central America	JN400552	JQ317621		X⑪
	108 *C. c. imitator*	CC55	UCR	10.35	-85.35	Palo Verde, E. MINAE, CR	Central America	JN409305	-----------	X	----
	109 *C. c. imitator*	CC02	UCR	10.08	-84.47	San Ramon, CR	Central America	JN409287	JQ317622	X	X⑪

**Species**	**Code**	**ID**	**Source**	**Lat.**	**Long.**	**Locality**	**Region**	**GenBank Cyt *b***	**GenBank D-loop**	**DS1**	**DS2**
	110 *C. c. imitator*	CC51	UCR	9.95	−84.55	San Mateo, Higuito, CR	Central America	JN409288	JQ317650	X	X⑪
	111 *C. c. imitator*	CU005	UCLA	9.78	−84.93	Curu, CR	Central America	JN409306	JQ317651	X	X⑪
	112 *C. c. imitator*	CC19	UCR	9.45	−84.15	Manuel Antonio, Quepos, CR	Central America	JN409307	JQ317652	X	X⑪
	113 *C. c. imitator*	J048	UCLA	9.73	−82.85	Cahuita, Limon, CR	Central America	JN409308	-----------	----	X⑪
	114 *C. c. imitator*	M004	UCLA	8.38	−83.28	Matapalo, CR	Central America	JQ317658	JQ317623	X	X⑪
	116 *C. c. imitator*	24825	LACM	10.26	-85.57	Santa Cruz, Guanacaste, CR	Central America	JN400550	-----------	----	X⑪
C. c. capucinus	117 *C. c. capucinus*	171487	USNM	9.48	−79.56	Cerro Bruja, PAN	Central America	JN409309	JQ317653	----	X
C. c. spp.	118 *C. c.* spp.	Genbank	GenBank	9.07	-79.55	Panama Zoo, PAN	Central America	AY065907	JQ317654	X	X
C. olivaceus brunneus	119 *C. o. brunneus*	372765	USNM	10.62	−68.41	Urama, Falcon, VEN	Coastal Venezuela	JN409289	JQ317624	----	X
	120 *C. o. brunneus*	443218	USNM	10.9	−68.77	Riecito, Falcon, VEN	Coastal Venezuela	JN409310	JQ317655	X	X
C. o. spp.	121 *C. o.* spp.	14378	LACM	9.74	−61.42	Barrancas, Delta Amacuro, VEN	Coastal Venezuela	JN409290	JQ317625	X	X⑩
C. o. apiculatus	122 *C. o. apiculatus*	296608	USNM	7.65	−66.17	Caicara, Monagas, VEN	North Amazon	JN409312	JQ317626	X	X

**Species**	**Code**	**ID**	**Source**	**Lat.**	**Long.**	**Locality**	**Region**	**GenBank Cyt *b***	**GenBank D-loop**	**DS1**	**DS2**
	123 *C. o. apiculatus*	388187	USNM	3.62	−65.68	Cerro Duida, AM, VEN	North Amazon	JN409313	JQ317627	X	X⑩
	124 *C. o. apiculatus*	388188	USNM	3.62	−65.68	Cerro Duida, AM, VEN	North Amazon	JQ317659	JQ317628	X	X⑩
	125 *C. o. apiculatus*	406451	USNM	7.5	−65.78	Santa Rosalia, VEN	North Amazon	JQ317666	JQ317656	----	X⑩
C. o. olivaceus	126 *C. o. olivaceus*	374805	USNM	6.29	−61.32	El Manaco, Bolivar, VEN	North Amazon	JQ317660	JQ317629	X	X⑩
	127 *C. o. olivaceus*	374796	USNM	6.29	−61.32	El Manaco, Bolivar, VEN	North Amazon	JN409291	JQ317630	X	X⑩
	128 *C. o. olivaceus*	449466	USNM	5.03	−60.95	San Ignacio de Yuruani, VEN	North Amazon	JN409314	JQ317631	X	X⑩
	129 *C. o. olivaceus*	374810	USNM	6.29	−61.32	El Manaco, Bolivar, VEN	North Amazon	JQ317661	JQ317632	X	X⑩
	130 *C. o. olivaceus*	443211	USNM	4.42	−61.58	Icaburu, Bolivar, VEN	North Amazon	JN409315	JQ317633	X	X⑩
C. o. castaneus	131 *C. o. castaneus*	339662	USNM	3.3	−58.88	Kuitaro, GUY	North Amazon	JN409316	JQ317634	X	X
C. o. nigrivitattus	132 *C. o. nigrivitattus*	CRB2440	GenBank	−0.96	−62.92	Barcelos, AM, BR	North Amazon	FJ529106	-----------	X	X⑩
	133 *C. o. nigrivitattus*	CRB2532	GenBank	−0.96	−62.92	Barcelos, AM, BR	North Amazon	FJ529107	-----------	X	X
	134 *C. o. nigrivitattus*	JPB OLIO	INPA	0.5	−64	Rio Negro, AM, BR	North Amazon	JN409335	JQ317635	----	X⑩
	135 *C. o. nigrivitattus*	JPB 130	INPA	0.85	−63.48	Barcelos, AM, BR	North Amazon	JQ317667	JQ317636	----	X⑩

**Species**	**Code**	**ID**	**Source**	**Lat.**	**Long.**	**Locality**	**Region**	**GenBank Cyt *b***	**GenBank D-loop**	**DS1**	**DS2**
	136 *C. o. nigrivitattus*	JPB OLIA	INPA	0.5	−64	Rio Negro, AM, BR	North Amazon	JQ317664	JQ317637	----	X⑩
Cebus albifrons spp.	137 *C. a.* spp.	Parna 066	PGLab	-4.66	-56.75	Itaituba, PNA, TPR, PA, BR	South Amazon	MF472591	MF472518	----	X
	138 *C. a.* spp.	398449	USNM	10.96	-74.79	Barranquilla, COL	North Andes	JN409318	JQ317643	X	X
	139 *C. a.* spp.	406628	USNM	3.17	-65.82	Tamatama, AM, BR	North Amazon	JN409320	-----------	----	X⑩
	140 *C. a.* spp.	136567	MVZ	-6.08	-75.06	Ucayali, PER	West Amazon	JN400553	-----------	----	X
C. a. cesarea	141 *C. a. cesarae*	27327	LACM	9.14	−73.57	San Alberto, Magdelena, COL	North Andes	JN409292	JQ317638	X	X
C. a. adustus	142 *C. a. adustus*	443501	USNM	9.2	−72.64	El Rosario, Zulia, VEN	North Andes	JQ317668	JQ317640	----	X
	143 *C. a. adustus*	443503	USNM	9.2	−72.64	El Rosario, Zulia, VEN	North Andes	JN409319	JQ317641	X	X
	144 *C. a. adustus*	443642	USNM	9.18	−72.7	El Rosario, Zulia, VEN	North Andes	JQ317662	-----------	X	----
C. a. pleei	145 *C. a. pleei*	281628	USNM	9.23	−74.42	El Rosario, Zulia, VEN	North Andes	JQ317669	-----------	X	----
C. a. leucocephalus	146 *C. a. leucocephalus*	443629	USNM	7.32	−71.96	Nulita, Apure, VEN	North Andes	JN409293	JQ317642	----	X

Species	Code	ID	Source	Lat.	Long.	Locality	Region	GenBank Cyt *b*	GenBank D-loop	DS1	DS2
C. a. trinitatis	147 *C. a. trinitatis*	24201	AMNH	10.39	−61.3	Tabaquite, Trinidad	Coastal Venezuela	JN409317	JQ317644	----	X
C. a. unicolor	148 *C. a. unicolor*	9922	LSUMZ	−10	−71.02	Rio Curanja, Balta, Loreto, PER	West Amazon	JN409295	JQ317645	X	X
	149 *C. a. unicolor*	193676	MVZ	−8.67	−72.78	Igarape Porongaba, Rio Jurua, BR	West Amazon	JN409323	JQ317649	X	X
	150 *C. a. unicolor*	406439	USNM	2.25	−65.28	Rio Macava, VEN	North Amazon	JN409321	-----------	X	X
	151 *C. a. unicolor*	CRB2678	GenBank	−0.96	−62.92	Barcelos, AM, BR	North Amazon	FJ529109	-----------	X	X
	152 *C. a. unicolor*	CRB1809	GenBank	−0.96	−62.92	Barcelos, AM, BR	North Amazon	FJ529108	-----------	X	----
C. a. yuracus	153 *C. a. yuracus*	F198	NYU	−0.7	−76.35	Yasuni, ECU	West Amazon	JQ317670	JQ317646		X
	154 *C. a. yuracus*	217	NYU	−0.7	−76.35	Yasuni, ECU	West Amazon	JN409322	JQ317657	X	X
	155 *C. a. yuracus*	153479	MVZ	−4.45	−78.27	AM, PER	West Amazon	JN409294	JQ317647	X	X
C. a. albifrons	156 *C. a. albifrons*	JPB 73	INPA	0.62	−65.92	Parque Nac. P. Neblina, AM, BRA	North Amazon	JQ317671	JQ317615		X
	157 *C. a. albifrons*	JPB 107	INPA	0.49	−65.27	Rio Negro, AM, BR	North Amazon	JQ317663	JQ317616		X
C. kaapori	158 *C. kaapori*	ckaa	PGLab	-3.01	-46.14	Maranhão, BR	Southeast Amazon	MF472590	MF472519	X	X

**Species**	**Code**	**ID**	**Source**	**Lat.**	**Long.**	**Locality**	**Region**	**GenBank Cyt *b***	**GenBank D-loop**	**DS1**	**DS2**
S. sciureus macrodon	Saimiri 1	GenBank	GenBank	UNK	UNK	UNK	UNK	HQ644338	---------	X	----
S. sciureus sciureus	Saimiri 2	GenBank	GenBank	UNK	UNK	UNK	UNK	HQ644334	---------	X	----
A. azarae	Aotus 1	GenBank	GenBank	UNK	UNK	UNK	UNK	KC757385	---------	X	----
A. azarae	Aotus 2	GenBank	GenBank	UNK	UNK	UNK	UNK	JN161099	---------	X	----
C. kuhlii	C. kuhlii	GenBank	GenBank	UNK	UNK	UNK	UNK	KR869628	---------	X	----
C. jacchus	C. jacchus	GenBank	GenBank	UNK	UNK	UNK	UNK	AB572419	---------	X	----
S. oedipus	Saguinus 1	GenBank	GenBank	UNK	UNK	UNK	UNK	KC757409	---------	X	----
S. oedipus	Saguinus 2	GenBank	GenBank	UNK	UNK	UNK	UNK	FJ785424	---------	X	----
L. rosalia	Leontopithecus	GenBank	GenBank	UNK	UNK	UNK	UNK	KC757399	---------	X	----
A. belzebuth	Ateles 1	GenBank	GenBank	UNK	UNK	UNK	UNK	FJ785422	---------	X	----
A. belzebuth	Ateles 2	GenBank	GenBank	UNK	UNK	UNK	UNK	KC757386	---------	X	----

**Species**	**Code**	**ID**	**Source**	**Lat.**	**Long.**	**Locality**	**Region**	**GenBank Cyt *b***	**GenBank D-loop**	**DS1**	**DS2**
A. seniculus	Alouatta 1	GenBank	GenBank	UNK	UNK	UNK	UNK	HQ644333	---------	X	----
A. caraya	Alouatta 2	GenBank	GenBank	UNK	UNK	UNK	UNK	KC757384	---------	X	----
L. lagotricha	Lagothrix	GenBank	GenBank	UNK	UNK	UNK	UNK	KC757398	---------	X	----
C. israelita	Chiropotes 1	GenBank	GenBank	UNK	UNK	UNK	UNK	KC592392	---------	X	----
C. albinasus	Chiropotes 2	GenBank	GenBank	UNK	UNK	UNK	UNK	KC757393	---------	X	----
P. pithecia	Pithecia 1	GenBank	GenBank	UNK	UNK	UNK	UNK	KR902426	---------	X	----
P. pithecia	Pithecia 2	GenBank	GenBank	UNK	UNK	UNK	UNK	KR902425	---------	X	----
C. cupreus	C. cupreus	GenBank	GenBank	UNK	UNK	UNK	UNK	KC959986	---------	X	----
C. donacophilus	C. donacophilus	GenBank	GenBank	UNK	UNK	UNK	UNK	FJ785423	---------	X	----

Country Codes: BR = Brazil; GUY = Guyana; COL = Colombia; PER = Peru; ARG = Argentina; PAR = Paraguay; HON = Honduras; CR = Costa Rica; PAN = Panama; VEN = Venezuela; ECU = Ecuador. **Institution Codes:** PGLab = Phylogenomics and Bioinformatics Laboratory of Universidade Federal do Pará; USNM = Unitad States National Museum; INPA = Instituto de Pesquisas da Amazônia, Brazil; AMNH = American Museum of Natural History; LSUMZ = Louisiana State University Museum of Zoology; UESC = Universidade Estadual de Santa Cruz, Brazil; CETAS[1] = Screening Center of Wild Animals, Salvador, Brazil; CETAS[2] = Screening Center of Wild Animals, Vitória da Conquista, Brazil; LACM = Los Angeles Country Museum of Natural History; U. Mon = University of Montana; CENP = Centro Nacional de Primatas, Brazil; UMMZ = University of Michigan Museum of Zoology; UCR = Universidad de Costa Rica; UCLA = University of California, Los Angeles; MVZ = Museum of Vertebrate Zoology, University of California; NYU = New York University. **Location Codes:** HPP = Hydroeletric Power Plant; PNA = Amazon National Park; SAJ = Santo Antonio do Jari; CP = Cachoeira de Porteira; REBIO = Biological Reserve.

River Codes: TR = Tucuruí River; XR = Xingu River; TPR = Tapajós River; JR = Jamari River; JAR = Jari River; UR = Uatumã River; TRR = Trombetas River. **Brazilian States:** PA = Pará; RO = Rondônia; AM = Amazonas; BA = Bahia; GO = Goiás; MG = Minas Gerais; ES = Espírito Santo; RJ = Rio de Janeiro; SP = São Paulo; MTG = Mato Grosso. **Word Abbreviations:** UNK = Unknown.
