# Supplementary material for: Phylogenetic relationships among Capuchin (Cebidae, Platyrrhini) lineages: An old event of sympatry explains the current distribution of Cebus and Sapajus
Source: Genet Mol Biol. 2018 Jul-Sep;41(3):699–712. doi: 10.1590/1678-4685-GMB-2017-0012 (PMC6136366; doi:10.1590/1678-4685-GMB-2017-0012)
Supplement: Supplementary file 2 [file 1415-4757-GMB-41-03-2017-0012-20180717-suppl3.pdf]

Supplementary Material to "Phylogenetic relationships among Capuchin (Cebidae, Platyrrhini) lineages: An old event of sympatry explains the current distribution of *Cebus* and *Sapajus*"

Table S2 – Characteristics of the datasets: final size, number of samples and models selected by PartitionFinder according to the program used in phylogenetic inferences.

**Datasets**	**Final Length (bp)**	**N. of samples**	**PF Model BEAST**	**PF Model RAxML**
DS1	946	80	HKY+I+G	-------------
DS2	1481	151	TrN+I+G	GTR+G+I

bp = Base Pairs; N. = Number; PF = PartitionFinder.
