# Supplementary material for: Phylogenetic relationships among Capuchin (Cebidae, Platyrrhini) lineages: An old event of sympatry explains the current distribution of Cebus and Sapajus
Source: Genet Mol Biol. 2018 Jul-Sep;41(3):699–712. doi: 10.1590/1678-4685-GMB-2017-0012 (PMC6136366; doi:10.1590/1678-4685-GMB-2017-0012)
Supplement: Supplementary file 3 [file 1415-4757-GMB-41-03-2017-0012-20180717-suppl5.pdf]

Supplementary Material to “Phylogenetic relationships among Capuchin (Cebidae, Platyrrhini) lineages: An old event of sympatry explains the current distribution of *Cebus* and *Sapajus*”

Methods Appendix S1 -The methodology of the BioGeoBEARS analysis with the four areas of Lima *et al.* (2017)

The biogeographical history and the ancestral area of the capuchins using the four area proposed by Lima *et al.* (2017) were made in the R package ‘BioGeoBEARS’ (Matzke, 2013, 2014). The same consensus tree used in the analysis with eight areas (see Material and Methods) was used here. The lineages were assigned to each of the four biogeographical areas defined following the known species distribution found in Rylands *et al.* (2013) in Amazonia (AM), Central America/Andes (CA), Atlantic Forest (AF) and Cerrado/Caatinga/Central Grasslands (CC) (see Lima *et al.* [2017] to understand the limit of each area).

A total of six models present in ‘BioGeoBEARS’ package was tested to explain the historical evolution of capuchin monkeys (DEC, DECJ, DIVALIKE, DIVALIKEJ, BAYAREALIKE and BAYAREALIKEJ). The lower estimated AIC value was used to choose the best model.
