# Supplementary material for: Phylogenetic relationships among Capuchin (Cebidae, Platyrrhini) lineages: An old event of sympatry explains the current distribution of Cebus and Sapajus
Source: Genet Mol Biol. 2018 Jul-Sep;41(3):699–712. doi: 10.1590/1678-4685-GMB-2017-0012 (PMC6136366; doi:10.1590/1678-4685-GMB-2017-0012)
Supplement: Supplementary file 4 [file 1415-4757-GMB-41-03-2017-0012-20180717-suppl4.pdf]

Supplementary Material to "Phylogenetic relationships among Capuchin (Cebidae, Platyrrhini) lineages: An old event of sympatry explains the current distribution of *Cebus* and *Sapajus*"

Table S3 – Comparison among the estimated models in 'BioGeoBEARS' considering the four areas proposed by Lima *et al*. (2017). For each implemented model in the analyses are shown: the log-likelihood values (LnL), number of parameters (n. params), dispersion (d), extinction (d), founder (j), and values of Akakie's Information Criteria (AIC and AIC weight).

	LnL	n. params	dispersion	extinction	founder	AIC	AIC weight
DEC	-18.056	2	0.026	10^{-12}	0.00	40.11	0.65
DEC+J	-17.666	3	0.025	10^{-12}	0.01	41.33	0.35
DIVALIKE	-18.706	2	0.038	10^{-12}	0.00	41.41	0.57
DIVALIKE+J	-17.998	3	0.031	10^{-12}	0.01	42.00	0.43
BAYAREALIKE	-23.623	2	0.037	1.46^{-1}	0.00	51.25	0.13
BAYAREALIKE+J	-20.737	3	0.028	10^{-07}	0.03	47.47	0.87
