# Supplementary material for: Phylogenetic relationships among Capuchin (Cebidae, Platyrrhini) lineages: An old event of sympatry explains the current distribution of Cebus and Sapajus
Source: Genet Mol Biol. 2018 Jul-Sep;41(3):699–712. doi: 10.1590/1678-4685-GMB-2017-0012 (PMC6136366; doi:10.1590/1678-4685-GMB-2017-0012)
Supplement: Supplementary file 5 [file 1415-4757-GMB-41-03-2017-0012-20180717-suppl1.pdf]

Supplementary Material to "Phylogenetic relationships among Capuchin (Cebidae, Platyrrhini) lineages: An old event of sympatry explains the current distribution of *Cebus* and *Sapajus*"

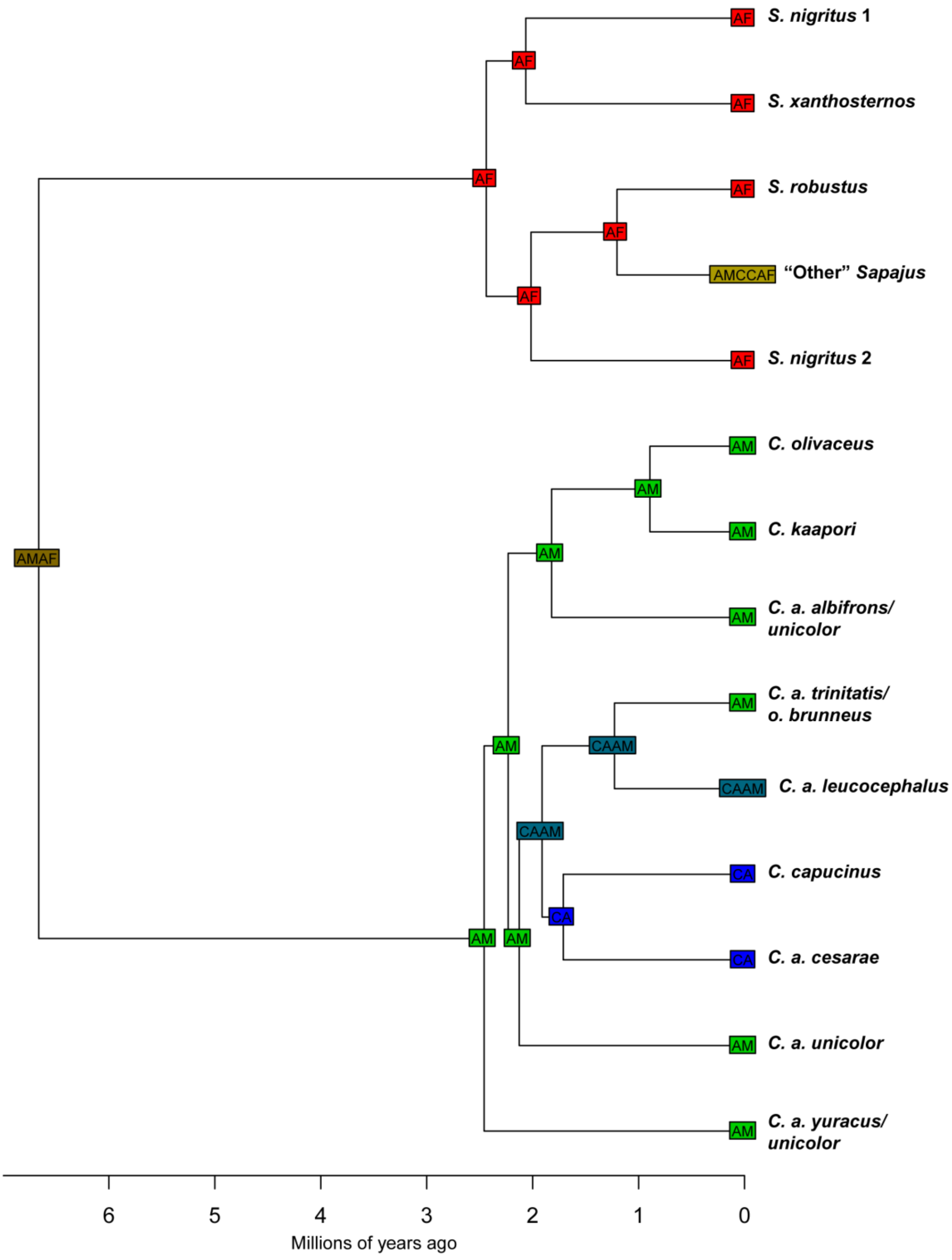

Figure S1 - DS2 time consensus tree with the estimates of ancestral areas of the capuchin monkeys considering the four areas proposed by Lima *et al.* (2017).
